# Illumination-Invariant and Deformation-Tolerant Inner Knuckle Print Recognition Using Portable Devices

**DOI:** 10.3390/s150204326

**Published:** 2015-02-12

**Authors:** Xuemiao Xu, Qiang Jin, Le Zhou, Jing Qin, Tien-Tsin Wong, Guoqiang Han

**Affiliations:** 1 School of Computer Science and Engineering, South China University of Technology, Higher Education Mega Center, Panyu, Guangzhou 510006, China; E-Mails: jin.q@mail.scut.edu.cn (Q.J.); z.le02@mail.scut.edu.cn (L.Z.); csgqhan@scut.edu.cn (G.H.); 2 National-Regional Key Technology Engineering Laboratory for Medical Ultrasound, School of Medicine, Shenzhen University, Shenzhen 518060, China; 3 Department of Computer Science and Engineering, The Chinese University of Hong Kong, Hong Kong 999077, China; E-Mail: ttwong@cse.cuhk.edu.hk; 4 Shenzhen Research Institute, The Chinese University of Hong Kong, Shenzhen 518057, China

**Keywords:** inner knuckle print recognition, illumination-invariant feature extraction, deformation-tolerant matching

## Abstract

We propose a novel biometric recognition method that identifies the inner knuckle print (IKP). It is robust enough to confront uncontrolled lighting conditions, pose variations and low imaging quality. Such robustness is crucial for its application on portable devices equipped with consumer-level cameras. We achieve this robustness by two means. First, we propose a novel feature extraction scheme that highlights the salient structure and suppresses incorrect and/or unwanted features. The extracted IKP features retain simple geometry and morphology and reduce the interference of illumination. Second, to counteract the deformation induced by different hand orientations, we propose a novel structure-context descriptor based on local statistics. To our best knowledge, we are the first to simultaneously consider the illumination invariance and deformation tolerance for appearance-based low-resolution hand biometrics. Settings in previous works are more restrictive. They made strong assumptions either about the illumination condition or the restrictive hand orientation. Extensive experiments demonstrate that our method outperforms the state-of-the-art methods in terms of recognition accuracy, especially under uncontrolled lighting conditions and the flexible hand orientation requirement.

## 1. Introduction

With the wide popularity of portable devices and ubiquitous computing, there is an increasing demand for a portable biometric system for authentication purposes. The cameras equipped on most portable devices are the most cost-effective devices for acquiring biometric information, without installing extra hardware components. Due to the relatively small and low-cost CCD or CMOS equipped in portable devices and the the uncontrollable acquisition conditions, the acquired images are easily contaminated with noise, blurriness, projection distortion, non-rigid deformation, cluttered background, as well as complex illumination. Hence, the major challenge is the robustness of the biometric system to all of these difficulties.

**Figure 1.**
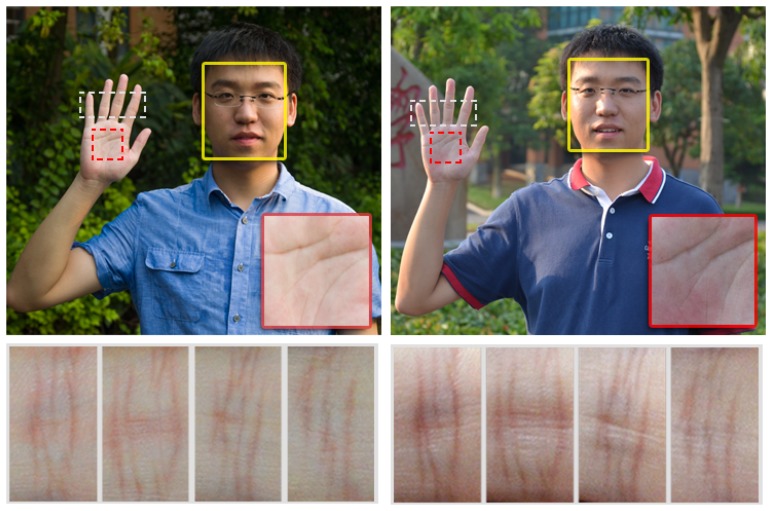
Pictures captured under different environments. The yellow, red and white boxes indicate the face, palm print and inner knuckle print (IKP) (middle nodes) characteristics, respectively. Note that the IKP's major structures are clear, rich and stable under different environments.

Existing portable, camera-based biometric systems utilize different biometric features. One popular stream is face recognition [1–5]. However, as demonstrated in the yellow boxes of Figure 1, the illumination cast on the faces can vary significantly. Besides, the facial expression and the aging factor of the subject further complicate the recognition. Many systems make various assumptions about the acquisition condition to ease the problem.

Another stream focuses on hand biometrics, including finger print, palm print, hand geometry, outer finger knuckle print (FKP) and inner knuckle print (IKP). This stream has received increasing attention recently, due to its user acceptability, hygienic concerns and easy self-positioning. Unfortunately, existing solutions remain quite limited. Finger print recognition [6] relies strongly on the quality of acquired images. High-quality image acquisition using a tailored high-resolution camera or scanner may be required. When using an ordinary camera for acquisition, sometimes only the crease structure of the palm print [7–10] (red boxes in Figure 1) can be extracted, which is insufficient for authentication purpose. Hand geometry features [11–13] also cannot uniquely identify a person's hand. These are usually combined with other biometric features. FKP recognition [14–18] is easily interfered by posture variation, and hence, a tailored device is usually required for achieving higher accuracy. On the other hand, IKP (the dashed white boxes in Figure 1), where the patterns are formed even before being born and determined by genes [19], is located on our fingers (geometrically close to cylinders). Its structure is clear and rich for uniquely identifying a subject. These properties allow IKP recognition to be valid under low-quality image acquisition, uncontrolled lighting conditions and positioning, and hence, it is promising as an alternative or supplement to other recognition measurements. Therefore, in this paper, we focus on IKP recognition.

Existing methods for IKP-based recognition can achieve high recognition accuracy (with an equal error rate (*EER* ) < 2.4%) under calibrated acquisition conditions [12,20–24]. However, the robustness of these methods under uncontrolled acquisition environments is questionable, mainly due to the following three issues.

- Low-quality image: Ordinary cameras on portable devices frequently introduce different noises when they are utilized to capture small image regions. As a result, the IKP structure in the image region is blurry, noisy and hard to extract.
- Illumination invariance: Illumination conditions are normally out of our control in our daily life. Changes in illumination lead to non-monotonic transformation of the intensity value and contrast, which may, in turn, lead to the weakening of important structures and magnifying the unwanted ones.
- Deformation tolerance: The flexible hand orientation would inevitably introduce two kinds of deformation due to the changes of posture and perspective viewing. Such deformations can significantly reduce the recognition accuracy.

In this paper, we propose a novel IKP recognition method that can operate in uncontrolled lighting conditions and that meets the more flexible hand orientation requirement. The illumination invariance is achieved by designing a novel feature detector, which can identify the major IKP structures well, while effectively suppressing the unwanted features (generated under uncontrolled acquisition environments) that may confuse the recognition and decrease the accuracy. The more flexible orientation requirement of our hands may introduce deformation due to illumination variance, perspective viewing and the change of posture. Deformation tolerance is achieved by applying a new structure-based context descriptor on our feature map. A block-based diagram is designed to reflect a rough local structure and to handle the aliasing problem due to the discrete nature of blocks and cells. Besides, a matching scheme is designed to perform histogram matching along both the orientation and the magnitude dimensions for revealing the true distance. Even though we only demonstrate the application of our method on IKP recognition, the proposed method is generic enough to be applicable to other applications, such as image quality assessment [25–27] and image retrieval [28].

As there are no public IKP databases available for benchmarking purpose, we built two databases, DB-1 and DB-2, corresponding to the calibrated and uncontrolled acquisition environment, respectively. Our experimental results show that, under similar acquisition environments (DB-1), our method can achieve comparable performance with state-of-the-art methods. On the other hand, when the acquisition environment is uncontrolled (DB-2), our method outperforms the state-of-the-art methods significantly. A high and stable recognition accuracy rate is achieved. Moreover, in the experiments, our method can tolerate sufficiently large deformation, and hence, the orientation of users' hands can be more natural during the acquisition. Our contributions can be summarized as follows.

- A novel illumination-invariant IKP feature extraction method taking both local and global information into account, which can highlight the major structures, while simultaneously suppressing the unwanted structure, hence remaining valid under uncontrolled illumination conditions.
- A novel matching algorithm that employs our structure-context descriptor to represent the structure and tolerate deformation, hence remaining valid under the more flexible and natural orientation of our hands.

The rest of this paper is organized as follows. Section 2 presents related work in the field of palm print, FKP and IKP. An overview of our system is given in Section 3. Section 4 describes the proposed illumination-invariant IKP feature extraction method. In Section 5, the deformation-tolerant matching algorithm is presented. The experimental results are reported in Section 6. The conclusion is drawn in Section 7.

## 2. Related Works

IKP recognition has only been introduced to biometric technology for a few years, but the methods for FKP and palm print may be also applicable for IKP, as all of them have similar line patterns. Currently, there have been some works that attempted to achieve robustness for illumination and deformation to some extent. We can classify these works into three categories.

A group of works were particularly designed for illumination robustness. Among them, the code-based approaches are popular, including CompCode [14,29], Oridinal Code [30], RLOC [31], phase code [16] and Riesz transforms [32]. However, the code maps in these methods seem likely to become unreliable in smooth regions when the illumination is varied nonlinearly. The local feature extraction approach is another research direction, including local binary pattern (LBP) [23,33,34], SIFT [35–37], phase congruency [16] and the magnitude of CompCode [14]. Nevertheless, as for SIFT, since IKP is usually composed of several line segments with few intersections, the features detected by SIFT are usually unreliable. As for LBP, it is sensitive to random noise and non-monotonic illumination variation, which often exist under an uncontrolled acquisition environment. The magnitude of CompCode is influenced by non-uniform illumination, since it is based on Gabor filtering, which is affected by the contrast values. As for phase congruency, although it is proposed to be invariant to illumination and can extract the major structures under different illumination, it also easily over-strengthens the unwanted structures under complex illumination. Therefore, currently we can hardly find any local feature extraction approaches that can reliably capture the IKP structures under different illumination conditions. Recently, some subspace methods have been employed to obtain the key structures by reducing the data dimension, including PCA [22,38] and LDA [39]. However, the capability of these methods to handle illumination and deformation is quite limited.

Another stream of works is focused on deformation tolerance. Most works are targeted at handling small deformations [15,17,18,21,40]. For example, Li *et al.* [21] proposed applying the Hausdorff distance, since it is an effective way to measure shape similarity and can take the deformation into account well. However, it has the risk of finding the wrong matches, since the pixel-based descriptor (e.g., position) may not be sufficient to correctly reflect the real structures. For example, one image containing two close lines and the other containing a single line would be considered very similar. There also some works attempting to handle pose invariance, by applying BLPOC [15,18], the reconstruction of homograph matrix [40], the reconstruction of the affine transformation [18] and image-based reconstruction [17]. However, BLPOC can only achieve translation invariance when images are captured in the same illumination, since it is sensitive to the change of illumination. The reconstruction of the homography matrix or the affine transformation matrix has the risk that the corresponding feature points may be unreliable, which quite possibly happens in IKP recognition, since IKP usually lacks valid feature points. Image-based reconstruction is a quite robust method, but it relies on a complete training database. However, this may be not so practical, since it may need enormous samples to cover all possible cases.

The last category collects the methods based on empirical models. For example, based on the prior knowledge that the IKP structure is usually a group of straight lines, Michael *et al.* [20] proposed to apply the ridgelet transform to extract IKP line features. Based on the prior knowledge that almost all of the IKP structure is vertically distributed, Li *et al.* [21] proposed applying a Radon transform to extract the line features, and Liu and Yan [41] proposed extracting the structure in the vertical orientation only. However, either ridgelet or Radon transform are influenced by illumination, since they do the statistics on intensity values. On the other hand, inspired by these works, we also integrated prior knowledge in our method and create a new model to strengthen the vertical structure and to suppress the horizontal structure.

Overall, existing methods attempted to achieve illumination robustness and deformation tolerance in a small range. Unfortunately, they are still far from satisfactory for the situation of uncontrolled acquisition environments.

## 3. System Overview

**Figure 2.**
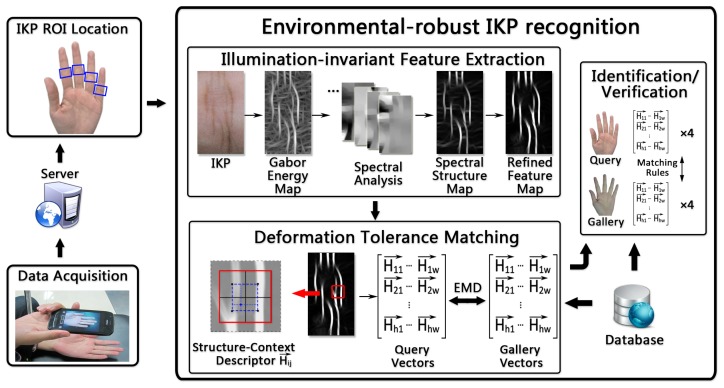
System overview of our identification system.

Figure 2 shows the pipeline of the proposed system, which is composed of three steps: data acquisition, ROI location, IKP recognition (including feature extraction and matching and hand identification/verification).

### 3.1. Data Acquisition

The hand images used in our system are acquired by ordinary cameras equipped on portable devices. We describe the uncontrolled acquisition environment as follows. First, the lighting condition and the camera exposure have no special restrictions, but there should be no severe over-exposure or under-exposure, as little recognizable structure information can be acquired in these two conditions. Second, the hand is asked to be naturally stretched out without any occlusion among the fingers. Third, the acquired image should cover the whole palm, and the palm should occupy the image as much as possible. Fourth, the background contains no color that is similar to the skin color. According to our experience, these restrictions are not tough and are easily satisfied for most portable devices in practice. The acquired hand images are then sent to a server via a wireless network.

### 3.2. ROI Extraction

**Figure 3.**
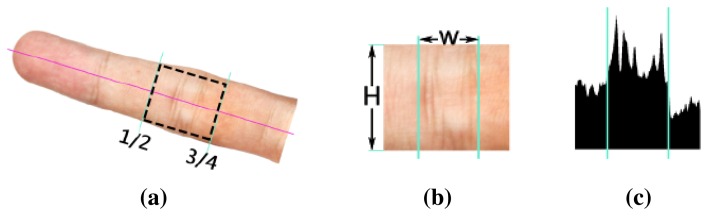
IKP ROI location: (**a**) ROI location; (**b**) normalization; (**c**) Radon projection.

We then extract the regions of interest (ROIs) of IKP from the images. In our system, we employ the IKP ROIs in the middle nodes of four fingers, except for the thumb (Figure 2), since their structures are relatively rich and clear. We first automatically separate the hand from the background by applying an integrated model of SCMand CASM [11]. Then, motivated by [12], the coarse ROI can be extracted based on the prior knowledge of the IKP geometry that the middle node is located between 1/2 to 3/4 the length of a finger (Figure 3a). This coarse ROI is rotated to a standard orientation by aligning the finger axis to the horizontal direction and resized to a unified scale according to a predefined height *H* (250 pixels in our implementation) (Figure 3b). Finally, the ROI is located using Radon projection, which covers the most informative areas in the projected result (Figure 3c) with a predefined length *W* (140 pixels in our implementation). As a result, all of the IKP ROI images are normalized to the same scale, rotation and translation.

### 3.3. IKP Recognition

To operate under the uncontrolled acquisition environments of portable devices, we propose an illumination-invariant feature extraction method tailored for IKP images. Furthermore, we propose a novel matching algorithm to accurately represent the structure and to tolerate deformations. We will detail them in Sections 4 and 5, respectively. As the IKP of each finger is highly unique and can be employed as a distinctive biometric identifier, one perfectly matched IKP is sufficient to authenticate a person. There is no need to match all four IKPs in an acquired image. For robustness, in our experiments, we take the following rules as our authentication criteria: two hand images are recognized as a matched pair when there is a perfect IKP matching pair or at least two fairly matched pairs. A perfect matching pair is defined as the case that the matching error is less than 0.8*T*, where T is the threshold and experimentally set as 0.41 in our work.

## 4. Illumination-Invariant Feature Extraction

Under uncontrolled acquisition environments, illumination variations easily lead to non-monotonic transformations of the gray scale, loss of details and ambiguous structures, making extracting features in an IKP image a difficult task. The aim of our method is to accurately extract the main features of an IKP image while eliminating the unwanted features that complicate the matching and recognition.

### 4.1. Local Feature Analysis

**Figure 4.**
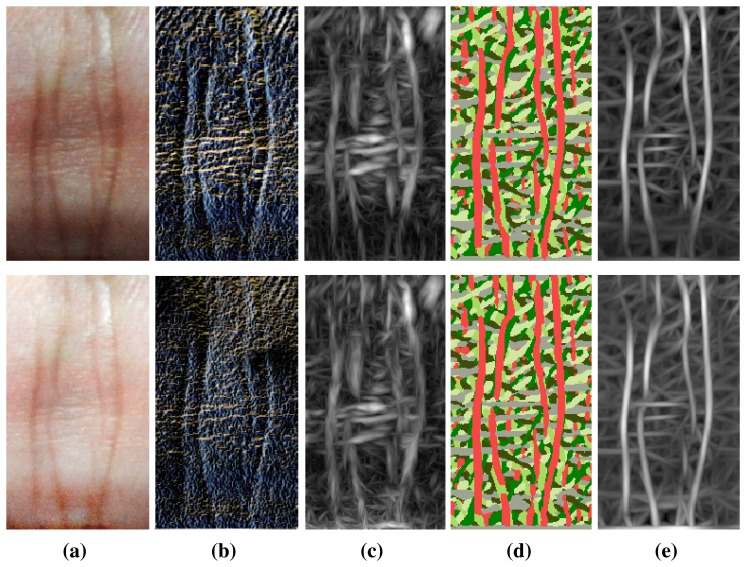
Existing feature detectors used for IKP images. (**a**) The IKP images under two illumination conditions; (**b**) the local binary pattern (LBP) maps; (**c**) the phase congruency (PC) maps; (**d**) the orientation; (**e**) the magnitude maps of CompCode.

In biometric systems, local feature detection methods have been extensively investigated. Figure 4 shows the results of some state-of-the-art local detectors aiming at illumination robustness, including local binary pattern (LBP) [23], phase congruency (PC) [29], a competitive coding scheme (CompCode) [16] and the magnitude map of CompCode [14]. In the IKP images (Figure 4a), there are a lot of fine lines around the main structures of the IKP, and they exhibit different characteristics under two different illumination conditions. Besides eliminating these unwanted structures, we have to avoid bringing in false structures caused by different illumination.

In the results of the LBP method (Figure 4b), a lot of week structures are detected with the main IKP structures, making robust matching of the IKP images challenging. This is because the LBP method is based on intensity distance, but the intensity distances for fine lines are locally large enough to be detected as features, especially under low lighting conditions. The PC method was originally proposed to extract the major structures under low contrast by searching the consistent phase regions. As a trade-off, PC often incorrectly strengthens weak structures at the same time, which is particularly serious for IKP images, as IKP images usually contain a lot of ambiguous structures, such as mutable creases and blood and meridian vessels. As shown in Figure 4c, these unwanted structures are also detected by PC. The CompCode method was proposed to obtain orientation information, so as to reveal the stable structure information under different illuminations. It performs well on regions containing major structures. However, the orientation values become unreliable and random on the smooth regions, since in these regions, their responses to different orientations are small, similar and, hence, vulnerable to noises, as shown in Figure 4d. Among these methods, it is observed that the magnitude of CompCode (Figure 4e outputs the best results, where the Gabor filtering is employed for the local statistics. It can reveal the local structures, while tolerating the monotonic illumination variation and small deformations. Hence, we adopt the CompCode magnitude map as our basic feature map, which is generated by:

(1) $$\Gamma \left(x,y,\lambda ,\theta_{j},\varphi ,\sigma ,\gamma \right)=\exp\left(-\left({x^{\prime }}^{2}+\gamma^{2}{y^{\prime }}^{2}\right)/2\sigma^{2}\right)\exp\left(i\left(2\pi x^{\prime }/\lambda +\varphi \right)\right)$$

(2) $$\text{Mag}\left(x,y\right)={\text{max}}_{j}\left\{\left|I\left(x,y\right)*\Gamma_{IM}\left(x,y,\theta_{j}\right)\right|\right\}$$

where Γ is a common form of the Gabor filter; *Mag*(*x*, *y*) is the magnitude map composed of the strongest response among different orientations for each pixel, and the response to each orientation is defined as the convolution of the preprocessed image *I* and the imaginary part of filtering Γ*_Im_*; *x′* = *x* cos *θ_j_* + *y* sin *θ_j_* and *y′* = −*x* sin *θ_j_* + *y* cos *θ_j_*; λ represents the wavelength of the sinusoidal factor; *θ_j_* is the orientation of the filters; *φ* is the phase offset; *σ* is the standard deviation of the Gaussian envelope and γ is the spatial aspect ratio. In our implementation, we set λ as 14, *φ* as 0, *σ* as 2π, γ as 0.5, *θ_j_* = *jπ*/*6* and *j* = 0,1, …, 5.

Although the magnitude maps of CompCode achieve good results for IKP images, it is noticeable that lots of weak and false structures still exist, which will greatly affect the accuracy of the following matching and recognition. As shown in Figure 4e, these unwanted features are widely distributed in the map, and some of them may have high energy values. In this case, it is difficult to use a local filter to remove these unwanted structures in the magnitude map.

### 4.2. Global Feature Extraction

To extract the main structures of IKP while suppressing the unwanted structures in the magnitude map of CompCode, we propose an efficient global-based algorithm. Inspired by the global detector proposed in [42], we first formulate the salient structure extraction problem as a partition of a weighted graph with the main structures of IKP as boundaries and then apply a spectral clustering algorithm to solve it. In this way, most false structures and weak structures caused by illumination variation across the main structures can be eliminated.

**Figure 5.**
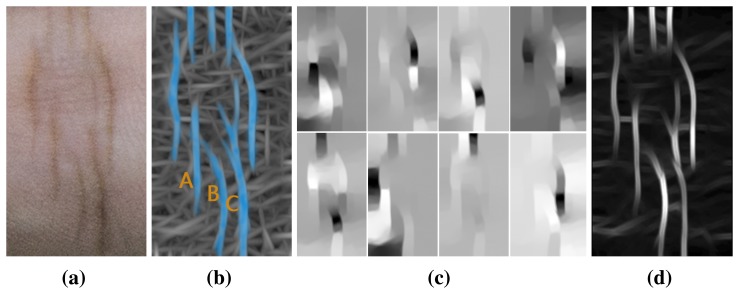
(**a**) The IKP image; (**b**) its magnitude map of CompCode can be divided into several regions (A, B, C, *etc.*) by the main structures of the IKP; (**c**) graphical illustration of eight non-zero eigenvalues; (**d**) the result of our global feature extraction algorithm.

It is observed that in the magnitude map of CompCode, the whole image can be divided into several regions by the main structures of IKP, as shown in Figure 5b. To that end, we can represent the map as a weighted graph, where every pixel corresponds to a node of the graph, and the relationship between two pixels corresponds to a weighted edge of the graph. The weights are calculated by a similarity matrix, which will be introduced later. Thus, to find the main IKP structure, we should find an optimal graph partitioning solution that ensures that pixels in each region of the partitioning solution have a high degree of affinity.

To reflect the degree of affinity between two pixels, we employ the idea of normalized cuts [43], based on spectral graph theory, to construct our detector. Its core idea is to construct a similarity matrix *W* and a Laplace matrix *D* − *W* to reflect the characteristics of the map and to obtain an approximate solution of multi-normalized cuts through solving a linear system. Then, we can construct the most salient features from the first several eigenvalues.

We construct a probability distribution of the boundaries of the map and define *P* as the probability of the boundaries. Then, we define the similarity matrix *W* as:

(3) $$W_{ij}=\exp\left(-\text{max}\left\{P\left(u\right)\right\}/\rho \right)$$

where *u* represents all pixels in the line from pixel *i* and *j*, and hence, *P*(*u*) is the probability that *u* is on the boundaries. In our implementation, we let *P* be equal to *Mag*(*x, y*) (see Equation (2) for its definition), which indicates the energy values of the structures. The larger the energy value, the greater the possibility that the pixel is on a boundary of the main structures. Considering the performance of the algorithm, we limit the the distance between *i* and *j* to eight pixels, and we set the parameter *ρ* to 0.1. To reflect global information, we define a diagonal matrix *D* based on *W_ij_*:

(4) $$D_{ii}=\sum_{j}W_{ij}$$

Thus, we can calculate the *n*+1 smallest eigenvalues 0 = λ_0_ ≤ λ_1_ ≤ … ≤ λ*_n_* and their corresponding eigenvectors {**v**_0_, **v**_1_,…, **v***_n_*}, which can describe the distribution information of IKP main structures, by solving the following linear system:

(5) (D-W)v=λDv

with the increase of *n*, the reliability of the eigenvalue to describe the distribution information is gradually reduced. Hence, we set *n* = 8 in our algorithm. Figure 5c shows the graphical illustration of the eight smallest non-zero eigenvalues.

We employ a standard spectral clustering algorithm to segment the graph, and the segmented boundaries embody the main structures of the IKP. For this purpose, we first calculate the directional derivative of n major eigenvectors to extract these structures and then compute the weighted summation of the structures:

(6) $$P_{s}\left(x,y,\theta \right)=\sum_{k=1}^{n}1/\sqrt{\lambda_{k}}\cdot \nabla \theta \upsilon_{k}\left(x,y\right)$$

where *P_s_* is the “spectral” component of the boundary detector, $1/\sqrt{\lambda_{k}}$ is the weight of the eigenvectors and ∇*_θ_v_k_*(*x*, *y*) represents the gradient in direction *θ*. Note that, different from the method proposed in [42], which aims at both the globally salient features and local edges in an image, we focus on extracting the main features from IKP images. In other words, we aim at eliminating all unwanted features for more robust recognition and are not trying to balance various scales of edges.

Although *P_s_* can provide the energy values for six orientations, generally, one dominant orientation is enough, since most of the IKP structures are straight lines. Hence, in our work, we use the dominant orientation and its energy to present the structures of IKP. We define the dominant orientation using a competitive rule as follows.

(7) $$\theta^{*}\left(x,y\right)=\text{argmax}\left\{(P_{s}\left(x,y,\theta \right)\right\},\theta =j\pi /6,j=0,1,\ldots ,5$$

Figure 5d shows the competitive energy map after performing our algorithm. Compared with Figure 4e, it is observed that most salient features are preserved, while a lot of weak and false structures are removed.

### 4.3. Refinement Using Empirical Model

**Figure 6.**
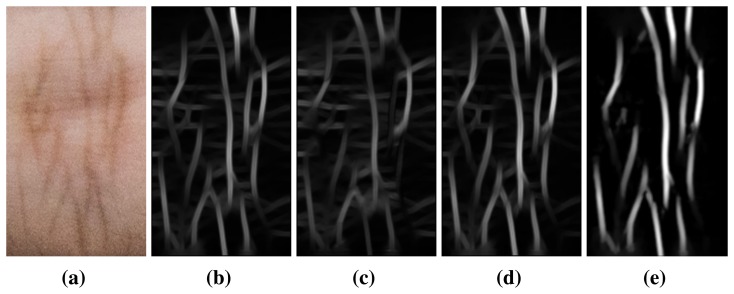
The feature refinement using our empirical model. (**a**) Original IKP image; (**b**) feature map after local and global extraction; (**c,d**) maximum energy maps based on (**b**) in the horizontal and vertical direction, respectively; (**e**) refined feature map.

Our global feature detection can remove most unwanted structures, but there are still some weak structures in the energy map, as shown in Figures 5d and 6b. After carefully studying the results, we find that most of these remaining weak features are fingerprint lines and small subcutaneous blood vessels in the horizontal direction, while the majority of reliable IKP structures are in the vertical direction. According to this observation, we refine our results by suppressing the weak structures in the horizontal direction while enhancing the structures in the vertical direction.

From Equation (6), we can obtain the probability distribution of IKP structures in all directions. We assume that when *θ* = 0°, 30°, 150°, the *P_s_* mainly represents the characteristics in horizontal directions, while when *θ* = 60°, 90°, 120°, the *P_s_* mainly represents the characteristics in vertical directions. In this regard, we refine our result using the following equation:

(8) $$P_{r}\left(x,y\right)=P_{s}\left(x,y,\theta^{*}\right)\cdot \text{min}\left\{1.0,{\left(P_{sv}\left(x,y\right)/P_{sh}\left(x,y\right)\right)}^{3}\right\}$$

where *P_sv_*(*x*, *y*) = *max_θ_*_=60°,90°,120°_ {*P_s_*(*x*, *y*, *θ*)} is the maximum of the vertical direction, *P_sh_*(*x*, *y*) = *max_θ_*_=0°,30°,150°_ {*P_s_*(*x*, *y*, *θ*)} is the maximum of the horizontal direction and θ* is the dominant orientation defined in Equation (7). Figure 6e shows the energy map after refinement. It is observed that after the refinement, the main structures are enhanced, and the unwanted structures are almost eliminated.

We employ a vector *A* to obtain a uniform feature representation for each pixel, which is composed of six components corresponding to the six orientations. After initialization, only the refined competitive energy values are stored in the vector, *A_xy_* [θ*/30°] = *P_r_*(*x*, *y*). The other five components are set to zero.

## 5. Deformation-Tolerant Matching Algorithm

Besides illumination variance, the uncontrolled acquisition mode would inevitably introduce deformations in the captured IKP images. Hence, deformation tolerance is another key issue that should be addressed for the success of an IKP recognition system.

The deformations can be classified into two categories: global deformations and local deformations. Global deformations are mainly caused by different hand postures, and we utilize the prior knowledge of the finger's geometry to obtain the coarse global normalization for the IKP ROI to address these posture-induced deformations. Please refer to Section 3 for details. In the following, we focus on local deformations, including non-rigid deformations and shear and perspective transformations.

**Figure 7.**
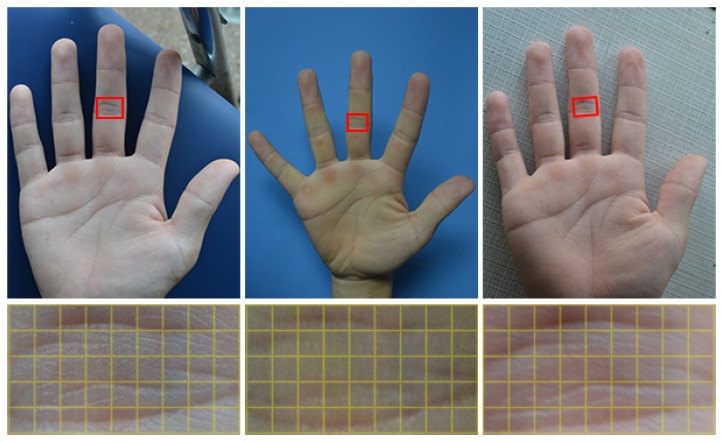
Three IKP images and their ROIs from the same finger node, but in different poses and viewpoints. The grids are used to show that the local deformations are often limited to small regions.

It is observed that the most local deformations are limited to a small region, as illustrated in Figure 7. In this regard, local statistics methods, such as the block diagram in HOG [44] or the log-polar diagram in shape context [45], should be sufficient to tolerate this kind of local deformation. On the other hand, sophisticated image mapping methods for dealing with big deformations, such as SIFT-based matching algorithms [35-37] or PTA [34], may fail with IKP images, as the IKP structures are often so simple that their eigenvectors are quite similar and lack uniqueness. Specifically, SIFT may fail to find sufficient reliable feature points, while PTA would easily find the wrong matched points. Therefore, we propose a new structure-context descriptor (SCD) performing local statistics on our feature maps to handle these local deformations.

**Figure 8.**
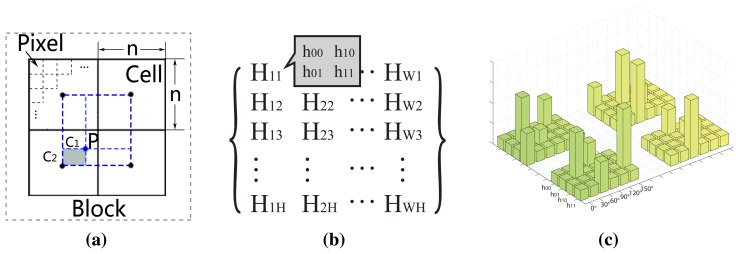
Deformation tolerance scheme: (**a**) structure-context descriptor. Each block has 2 × 2 cells, and each cell has *n* × *n* pixels, the set of which has 16 empirically in our work; (**b**) matrix of the histograms for the feature map; W and H in subscripts represent the width and height of the input image, respectively; (**c**) 3D model to present part of the feature map in (b). Each 4*d* × 6*bin* histogram corresponds to a point in vector *A*.

Our descriptor is designed to reflect the structure context in the neighborhood well, while tolerating small local deformation. Actually, different diagrams can be used for our descriptor [44,45]. However, to simplify the computation, motivated by HOG [44], we apply the block diagram to our feature map, as shown in Figure 8a. For each cell, we summarize all of the feature vectors of the pixels in this cell to reflect its rough structure. In this way, for one cell, the feature vectors of pixels are accumulated, and this leads to its nature of deformation tolerance. In other words, the degree of deformation tolerance is implicitly defined in the cell size. However, due to the discrete nature of the cell and block, an aliasing problem inevitably occurs. To circumvent this problem, as shown in Figure 8a, we first assign the value of each pixel to its nearest four cell centers by bilinear interpolation; then, we broaden half of a cell along the boundary of the block, as the pixels in this extended region would also contribute to the histogram of the cell. Thus, for a block diagram, the histogram of each cell can be calculated by:

(9) $$h_{kt}=\frac{1}{n^{2}}\sum_{i=\left(-\frac{3}{2}+k\right)n}^{\left(\frac{1}{2}+k\right)n}\sum_{j=\left(-\frac{3}{2}+t\right)n}^{\left(\frac{1}{2}+t\right)n}C_{1}\cdot C_{2}\cdot A_{xy}$$

where *n* is the length of a cell, $C_{1}=n-\left|\frac{1}{2}n+{\left(-1\right)}^{k}\cdot i\right|$. As shown in Figure 8a, *C*_1_ and *C*_2_ represent the length and width of the shaded rectangle, respectively, and their product represents the area of the rectangle. {*h*_00_, *h*_10_, *h*_01_, *h*_11_} are the histograms of the four cells in a block, and they are integrated sequentially to form the complete histogram *H* for a block consisting of 24 components; *x* = *i* + *x*_0_, *y* = *j* + *y*_0_, where (*x*_0_, *y*_0_) is the coordinate of the block center.

Next, we apply our SCD on our feature map, scanning the whole map by moving the block diagram from left to right and top to bottom, with a stride of 0.5*n*. After that, a matrix of histograms is produced to describe the feature map, as shown in Figure 8b,c.

**Figure 9.**
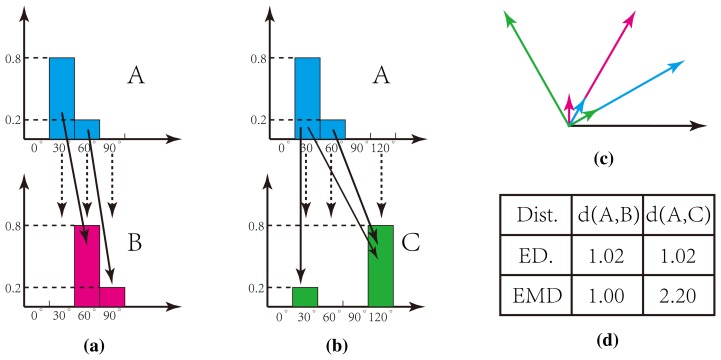
Measurement of the histogram distance by Euclidean and Earth mover distance (EMD).

The measurement of the histogram distance for our SCD is crucial for successful recognition. Euclidean distance, however, fails to reflect the true distance. As shown in Figure 9, it is clear that histograms *A* (blue vector in (c)) and *B* (green vector in (c)) are similar, while *A* and *C* (red vector in (c)) are much more different. However, these two groups are evaluated with the same distance based on the Euclidean distance, which are indicated by the dashed lines in Figure 9a,b. To tackle this problem, we adopt the Earth mover distance (EMD) [46], in which both the orientation and energy value can be taken into account, and an optimal solution will be found by minimizing the movement cost, as indicated by the solid lines in Figure 9a,b. Thus, the EMD distance can evaluate the true distance of our histograms, as shown in Figure 9d. Therefore, the distance of two images *D*(*S*, *S*′) can be calculated by:

(10) $$D\left(S,S\prime \right)=\sum_{i=1}^{N}\sum_{j=1}^{M}d\left(H_{ij,}H_{ij}^{\prime }\right)$$

(11) $$d\left(H,H\prime \right)=\sum_{k=0}^{1}\sum_{t=0}^{1}D_{\text{EMD}}\left(h_{kt},h_{kt}^{\prime }\right)$$

where *d*(*H*, *H*′)is the histogram distance of two blocks using EMD; *S* and *S*′ are the two compared KP images; *N* and *M* are the number of block diagrams applied in the *x* and *y* directions, respectively

## 6. Experimental Results

### 6.1. IKP Database and the Test Protocol

**Figure 10.**
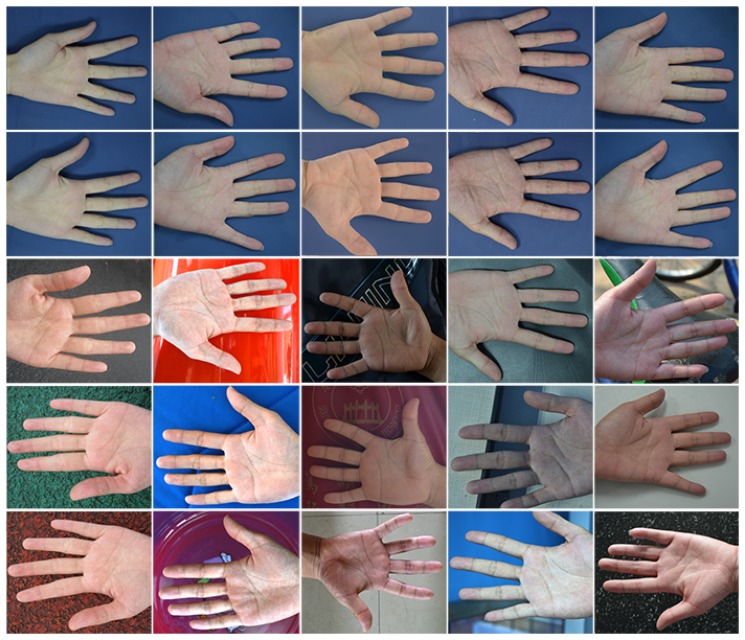
Samples in our IKP database. Each column corresponds to one hand.

To the best of our knowledge, currently, there is no publicly available IKP database for benchmarking. To evaluate the performance of our proposed system, a specific database was built by us. Our IKP images were collected from 100 volunteers, including 60 males and 40 females. Among them, 80 subjects were 18–30 years old, and the others were 30–52 years old. For each subject, five images of each hand were captured, corresponding to each column in Figure 10. Two of them were from the same environment with similar postures (the top two rows in Figure 10), and three of them were from different real environments with relatively large variations of posture (the lower three rows in Figure 10. Thus, there are 1000 hand images and 4000 extracted ROIs available in total.

To better compare our proposed method with existing methods in terms of the robustness to illumination variance and deformation, we divided our IKP images into two groups, DB-1 and DB-2. DB-1 contains only the IKP images captured under the same environments, two images for one hand (top two rows in Figure 10), 400 images and 1600 ROIs in total. DB-2 contains the IKP images captured under different environments, four images for one hand, 800 images and 3200 ROIs in total.

We measure the performance in terms of recognition accuracy using both verification and identification modes. Generally, identification is a one-to-many comparison against all stored templates, which answers the question “Which one of N people is this person?”. In the identification experiments, the statistics, best identification rate (BIR), which is known as the rank-1 recognition rate, as well, is adopted for evaluation. Verification is a one-to-one comparison against a single stored template, which answers the question of “whether the person is whom he claims to be”. In the verification experiments, the statistics, equal error rate (EER), is adopted to evaluate the performance. EER is defined as *EER* = *FAR* when the false acceptance rate (FAR) is equal to the false rejection rate (FRR). Here, *FAR* = *FA*/(*GR* + *FA*), *FRR* = *FR*/(*GA* + *FR*), and FA, FR, GA and GR are the proportions of false acceptances, false rejections, genuine acceptances and genuine reflections, respectively. In general, the lower the EER value is, the more reliably the biometric system performs.

### 6.2. Comparison with Existing Methods

We compare our method with state-of-the-art methods, including ImCompCode + MagCode [14 LGIC2 [16], R-ABF [17] and local block BLPOC [18], respectively. Although these four methods are not directly proposed for IKP recognition, they are currently the most effective methods for the recognition of IKP, FKP and palm print and are targeted at achieving robustness to illumination and deformation. We conduct the experiments on DB-1 and DB-2, respectively.

Experiment on DB-1: We start from the single-ROI recognition, which means the recognition unit is a single ROI image. In this case, the identification test will be carried out 1600 times, and the verification test will be performed 1600 × 1599 times. Next, we perform integrated ROI recognition, where the final decision is made based on the four ROIs' matching results from the same hand image. In this regard, verification is processed 400 × 399 times and identification 400 times.

Table 1 illustrates the performance of all methods based on DB-1 in two modes of both single ROI recognition and integrated ROI recognition. As for single ROI recognition, note that the results of all methods are acceptable for a biometric security system, and our method has better recognition accuracy than others. The accuracy is further improved if integrated ROI recognition is applied, since three more matching results can be used for making the final decision. Table 1 proves that all of the methods are robust to the small variations of illumination and deformation.

**Figure 11.**
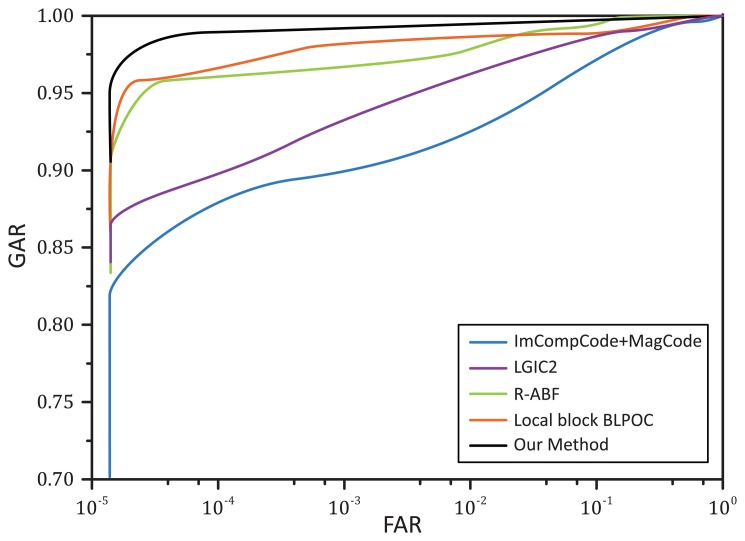
The ROC curves of all methods for single ROI recognition based on DB-1.

Figure 11 illustrates the receiver operating characteristic (ROC) curves, which plot the false acceptance rate (FAR) against the genuine acceptance rate (GAR). Here, we only plot the curves for single ROI recognition, since the results for integrated ROI recognition are too close to be separated clearly. For a biometric security system, the false acceptance is usually much more unacceptable than the false rejections. Hence, for a good biometric system, its GAR value should increase very quickly against the FAR value. From Figure 11, it is noticeable that, according to the same value of FAR, our method can always achieve the highest GAR, which implies that our method has better recognition accuracy.

Experiment on DB-2: Similar to experiments on DB-1, we also carry out the single ROI and integrated ROI recognition on DB-2.

Table 2 illustrates the performance of all methods based on DB-2. As for single ROI recognition, it is obvious that the existing methods have EER values larger than 7.0, which are far from acceptable for a biometric security system. Even for the integrated-ROI recognition, their results are larger than 2.26, which is also unacceptable. However, our method can obtain 3.07 and 0.74 for single ROI and integrated ROI recognition, which should be quite acceptable for a biometric system.

We found that the method of local block BLPOC has the worst result. That is because the features extracted by BLPOC are not robust to the contrast variations caused by illumination or deformation. ImCompCode improved the original CompCode scheme by proposing an evaluation method for detecting the “plane” pixels and removing them from orientation coding. However, it usually fails under complex illumination, since sharp edges may be smoothed and weak edges are strengthened. LGIC2 proposes to combine multiple features, including ImCompCode, phase congruency and phase code. Unfortunately, it is also unstable under complex illumination and easily strengthens the unwanted structures. R-ABFaims at solving the problem of posture deformation, but it cannot handle illumination variances and requires a database that should cover most of the possible postures. However, our database only contains five postures for each hand, and it is difficult to construct a database that fulfills the requirement of R-ABF.

**Figure 12.**
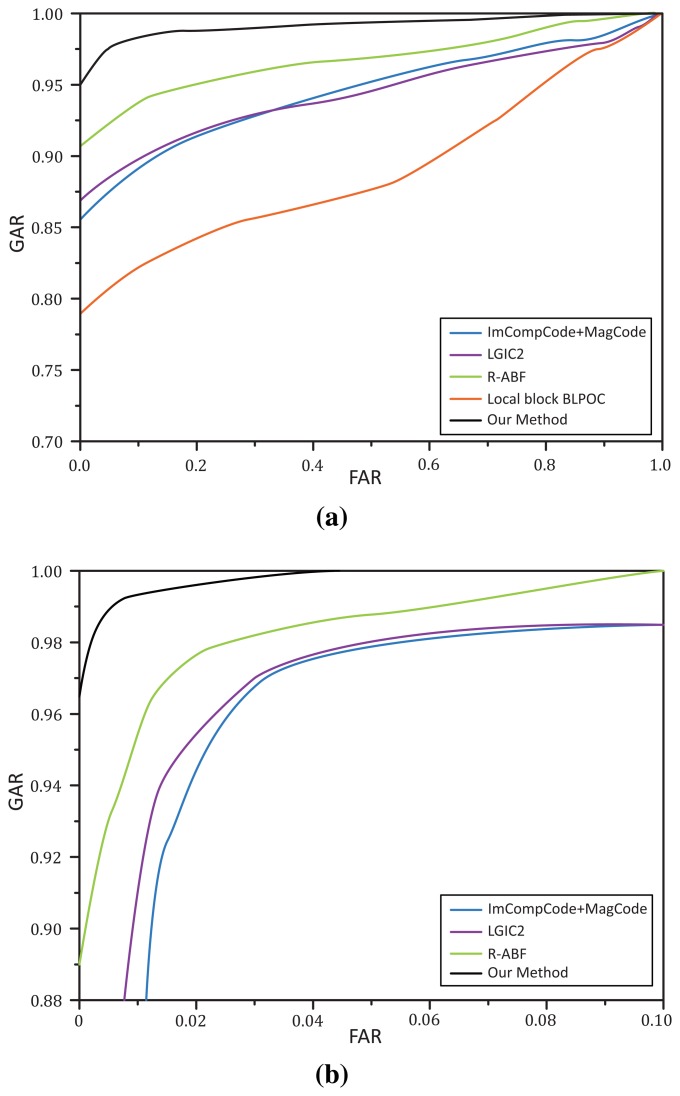
The ROC curves of all methods based on DB-2. (**a**) Single ROI recognition; (**b**) Integrated ROI recognition.

In our work, we propose a tailored feature extraction scheme for IKP images, which can almost operate under different illumination. Figure 12 illustrates the receiver operating characteristic (ROC) curves for single ROI and integrated ROI recognition, respectively. According to the same value of FAR, our method can always achieve a much higher GAR than others, which implies that our method has better recognition accuracy.

### 6.3. Further Discussions

#### 6.3.1. Threshold Determination

**Figure 13.**
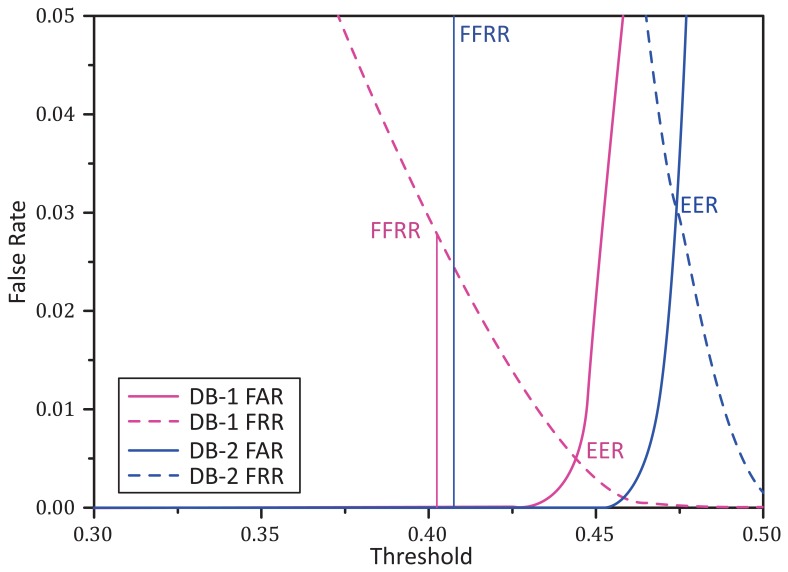
The curves of the false acceptance rate (FAR) and false rejection rate (FRR) against the threshold for DB-1 and DB-2. Only single ROI recognition is carried out here, since it can better reflect the performance of our matching algorithm.

For a biometric security system, a unified reliable threshold is necessary for recognition. Unfortunately, there is no convincing method to determine the threshold in this kind of system. Thresholds corresponding to EER have been used in some cases for this purpose. However, as shown in Figure 13, thresholds on EER are unstable and varied largely under the two databases. Considering that the target threshold is used for separating the matched and unmatched cases, thresholds corresponding to the minimum mismatching should approximate the true threshold. We define the minimum mismatching as the fewest false recognitions rate (FFRR), calculated as *FFRR* = *min* [(*FA* + *FR*)/(*GA* + *GR* + *FA* + *FR*)], where *FA*, *FR*, *GA*, *GR* indicate false acceptance, false rejection, genuine acceptance and genuine rejection, respectively. Figure 13 shows that thresholds for the FFRR are much more stable under the two databases compared to the thresholds for EER. Hence, in our system, we apply the threshold of the FFRR for determining matching or not. In practice, we set the threshold to 0.41.

#### 6.3.2. Tolerance to Deformation

**Figure 14.**
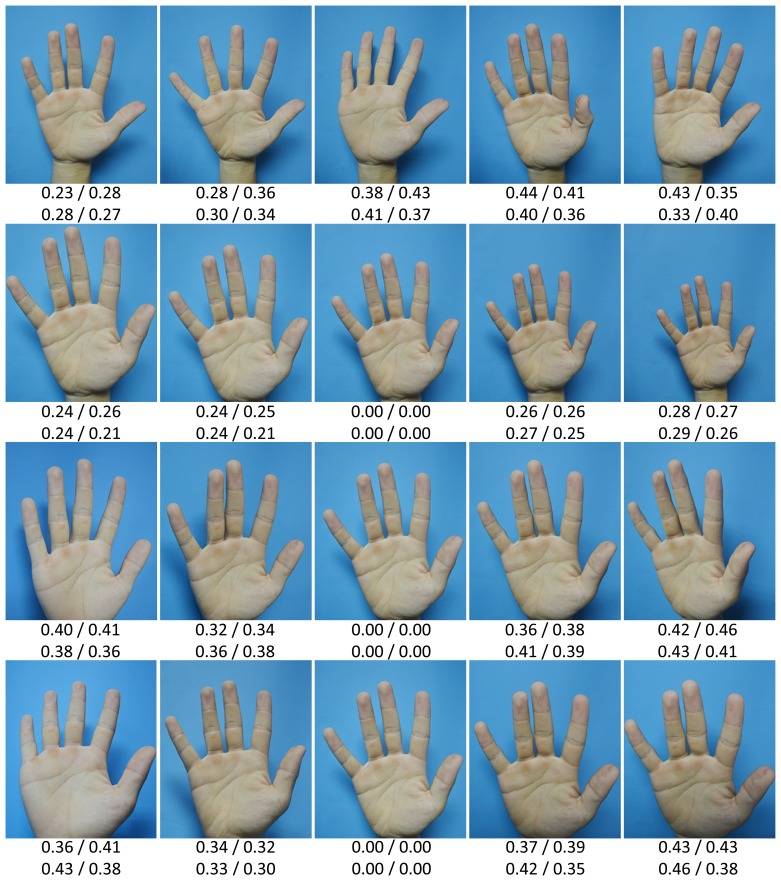
Samples of the deformed IKP images that can be tolerated by our method. From top to bottom: different postures, viewpoints from near to far, viewpoints from left to right, viewpoints from bottom to top. The four values under each image indicate the matching distances of the four IKP ROIs respectively. The threshold applied here *T* = 0.41 is the threshold corresponding to the FFRR from DB-2, and the matching decision is made based on integrated ROI recognition.

**Figure 15.**
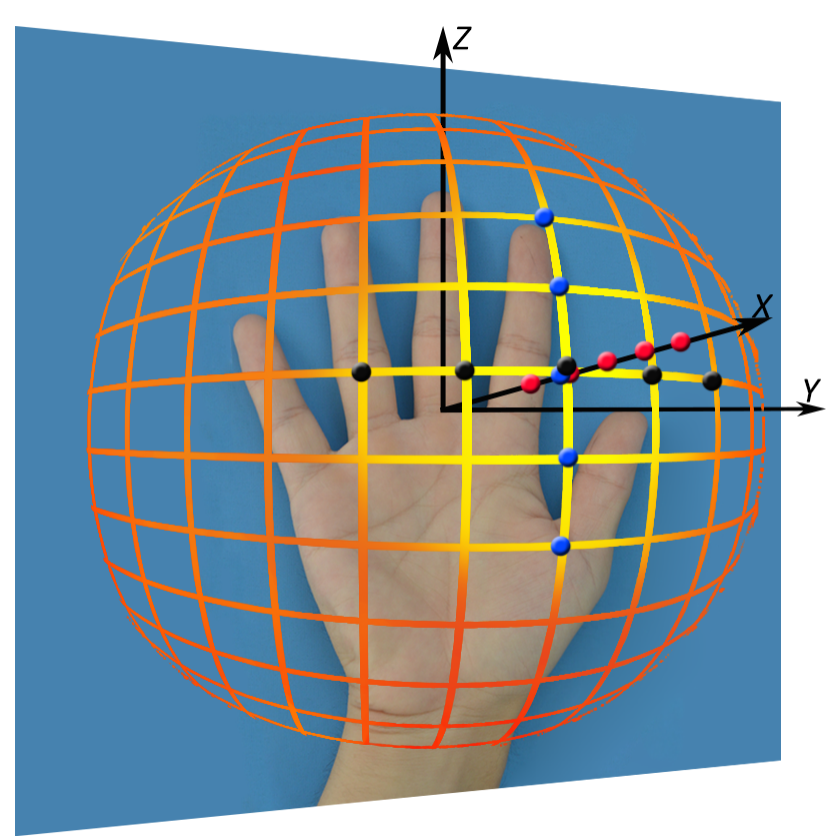
The simulation of viewpoint variation. The yellow region indicates the range that our method can tolerate. The images captured at the red, black and blue points are shown in the second to fourth row in Figure 14 respectively.

To validate the deformation tolerance of our method, we prepare the test samples in two ways: changes in posture (the top row in Figure 14) and viewpoints (Figure 15 and the lower three rows in Figure 14).

As for the deformation due to changes in posture, we can find that once our loose restrictions on acquisition (specified in Section 3.1) are satisfied, almost all of the posture variations can be well tolerated by our system. The first row in Figure 14 shows some challenging cases by changing the postures, and all of them can be successfully recognized by our method. As for the deformation due to the change of viewpoints, we use five concentric spheres to approximate the variation of viewpoints, as shown in Figure 15. The radius for the smallest sphere is defined as *r*, which is actually the distance between the hand and camera, and it is determined once the hand can almost fill the whole image. The radii for the other spheres are defined as 1.2*r*, 1.4*r*, 1.6*r* and 2.0*r*, indicated as the five red points on the *x* axis. Moreover, we further sample the viewpoints on each sphere uniformly with a step of 15 degrees. In Figure 15, we only show the uniform sampling viewpoints for the sphere with 1.2*r* for the purpose of clear visualization. The two sequences of black and blue points indicate that the viewpoints changed from left to right and from bottom to top, respectively. Based on our experimental results, our method is invariant to the viewpoints on the *x* axis, and it can endure 30° (yellow region in Figure 15), referring to the *x* axis. Here, we define the *x* axis as the vector going through the center of the hand (the root of the center finger) and facing the hand plane perpendicularly. Figure 14 shows some challenging IKP images that can be tolerated by our method, demonstrating the capability of our method to tolerate deformations.

#### 6.3.3. Tolerance to Illumination Variation

**Figure 16.**
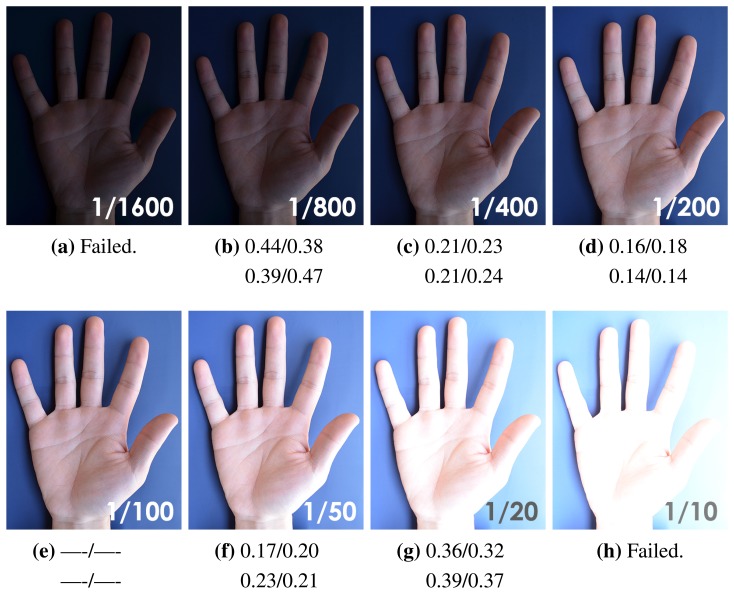
Comparison under different exposures. All images are captured by a Nikon D7000 camera with a F5.0 aperture. The values located in the right bottom corner of the images are the exposure time (in seconds). The four values under each image indicate the matching distances of the four IKP ROIs against (e), which is considered to be under perfect illumination conditions, respectively. The threshold applied here is 0.41, the same as in Figure 14.

Tolerance to illumination variation plays a crucial role in our biometric recognition system. In order to measure the performance of our method under different illumination, we capture samples under several scenes and analyze the matching distances. We first change the shutter speed to simulate the illumination variation in the same scene. Figure 16 shows part of the captured images with the shutter speed varied from 1/800 s to 1/20 s. Note that our method has decent robustness to the exposure variation, unless the structures of the IKP are permanently lost due to overexposure or underexposure (Figure 16a,h).

**Figure 17.**
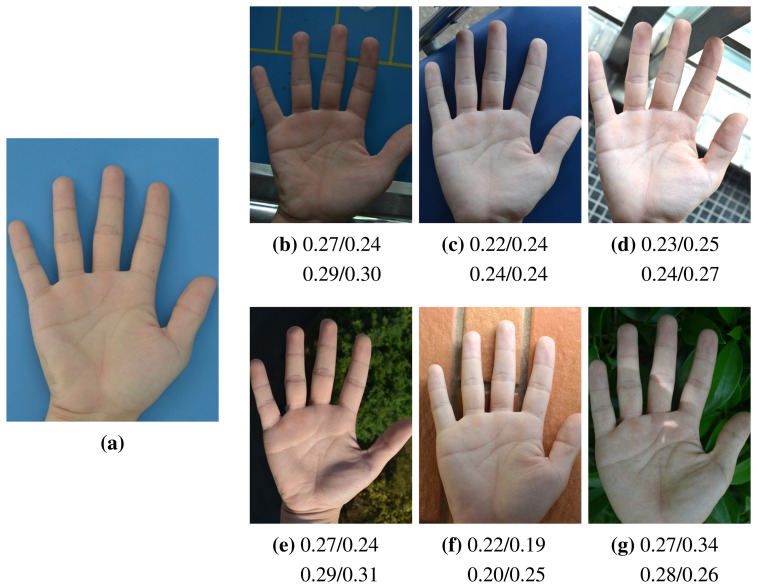
Some examples in typical scenes. (**a**) Ideal experimental environment; (**b**–**d**) indoor scenes under different illumination; (**e**) scene under direct sunlight; (**f**) scene under side light; (**g**) scene under non-uniform illumination and shadow. The four values under each image indicate the matching distances of the four IKP ROIs against (a), respectively. The threshold applied here is 0.41, the same as in Figure 14.

Next, the influence of ambient light variation is considered in the following experiment. Since ambient light is composed of complex direct light and reflected light, it is difficult to quantize. We utilize a few typical scenes to simulate different settings of ambient light and evaluate the tolerance of our method to them. Figure 17 shows that our system performs robustly under different scenes, which implies that our method has decent tolerance under random illumination.

#### 6.3.4. Time Performance

We implement our system, as well as the other four competitors on a PC with Intel Core i7 4790 K 4.4 GHz CPU with eight threads, 16 GB RAM and the Microsoft Windows 7 64-bit operating system. Table 1 shows that all of the methods achieve high efficiency, although our method is a little slower than the others. Meanwhile, our method can maintain high efficiency even under an enormous database, since the features of the training images are extracted offline and only the matching algorithm is computed online, which is so efficient due to parallel computation (generally < 50 ms for one thousand times).

## 7. Conclusions

We propose a robust IKP matching and identification method for biometric authentication purposes. The proposed method can survive under uncontrolled lighting conditions, noisy and blurry image acquisition, as well as deformation due to hand orientation and perspective projection. Hence, users of the biometric authentication system can be relieved from the careful planning of the acquisition environment and the unnatural hand orientation during the capture. The proposed method outperforms state-of-the-art biometric feature recognition methods when the acquisition environment is challenging. Our current algorithm still requires a remote server for execution due to the relatively high computational cost. We believe that, by redesigning and simplifying the algorithm, the method should be lightweight enough to be executed on portable devices without a remote server.

## Figures and Tables

**Table 1.** Comparison of the five methods based on Database 1 (DB-1) in terms of accuracy and time performance. Note that “Sin.-ROI” and “Int.-ROI” are the abbreviations of single ROI and integrated ROI recognition, respectively. Here, we compute the time that is spent for verification or identification for one test dataset in integrated ROI mode. BIR, best identification rate; EER, equal error rate.

	**Identification BIR (%)**		**Verification EER (%)**		**Time (ms)**	

	**Sin.-ROI**	**Int.-ROI**	**Sin.-ROI**	**Int.-ROI**	**Verifi.**	**Identi.**
ImCompCode + MagCode	90.90	100	2.06	0.233	110	145
LGIC2	94.5	100	1.81	0.207	204	250
R-ABF	97.3	100	1.25	0.134	223	279
Local block BLPOC	98.3	100	1.23	0.078	187	234
Our method	100	100	0.50	0.021	671	768

**Table 2.** Comparison of the five methods based on DB-2. Note that “Sin.-ROI” and “Int.-ROI” are the abbreviations of single ROI and integrated ROI recognition, respectively.

	**Identification BIR(%)**		**Verification EER(%)**	
	**Sin.-ROI**	**Int.-ROI**	**Sin.-ROI**	**Int.-ROI**
ImCompCode + MagCode	83.3	97.0	11.23	3.09
LGIC2	86.6	98.5	10.13	3.04
R-ABF	85.6	96.2	7.14	2.26
Local block BLPOC	76.3	90.8	26.75	14.67
Our methods	93.5	100	3.07	0.74
